# Supplementary material for: Community perceptions of yellow fever and its treatment practices in regions with reported outbreaks in Uganda: A qualitative study
Source: PLoS Negl Trop Dis. 2025 Jul 14;19(7):e0013251. doi: 10.1371/journal.pntd.0013251 (PMC12270305; doi:10.1371/journal.pntd.0013251)
Supplement: Attachment — Submitted filename: Letter_YF-Perception_Response_to_Reviewers.pdf [file pntd.0013251.s003.pdf]

Dr. med. univ. Lena Huebl
Department of Tropical Medicine,
Bernhard Nocht Institute for Tropical Medicine
& I. Department of Medicine,
University Medical Center Hamburg-Eppendorf,
Bernhard-Nocht-Str. 74
D-20359 Hamburg, Germany
Phone: +49 40 42818-0
Email: lena.huebl@bnitm.de
Web: www.bnitm.de

Hamburg, 24th May 2025

Dear Editors,

We would like to thank the reviewers for their helpful suggestions, which improved the quality of manuscript PNTD-D-25-00279, “Community Perceptions of Yellow Fever and its Treatment Practices in Regions with Reported Outbreaks in Uganda: A Qualitative Study.”

We revised the manuscript based on the reviewer’s comments. We uploaded a point-by-point response to the reviewer’s comments. We uploaded two versions of the revised manuscript: a clean version and a version with changes highlighted in yellow.

We hope you will consider our revised article for publication in your Journal.

Sincerely,

Lena Hübl

Lena Huebl

Lena Huebl, MD, DTM&H
Resident in internal medicine, infectious diseases, and tropical medicine
PhD candidate in Public Health

## Response to Reviewer

**Key Review Criteria Required for Acceptance?**
As you describe the new analyses required for acceptance, please consider the following:

**Methods:**
-Are the objectives of the study clearly articulated with a clear testable hypothesis stated?
-Is the study design appropriate to address the stated objectives?
-Is the population clearly described and appropriate for the hypothesis being tested?
-Is the sample size sufficient to ensure adequate power to address the hypothesis being tested?
-Were correct statistical analysis used to support conclusions?
-Are there concerns about ethical or regulatory requirements being met?

Reviewer #1:

1. **Methodological Clarity**
   - Specify the average interview duration.
     Thank you, we added this information, now in line 205.
   - Provide evidence of interview guide validation.
     Thank you, we added this important information, now in lines 195-198.
   - Clarify sampling strategy (purposive sampling, snowball sampling)
     Thank you, we used purposive sampling. This has been stated in line 168.
   - Explain the method for determining interview saturation.
     Thank you, we added this information, now in lines 206-208.
2. **Research Rigor**
   - Conduct researcher triangulation to enhance methodological reliability.
     Thank you, we added this information, now in lines 198-202.
   - Clearly delineate novel insights beyond existing theoretical frameworks
     Thank you for pointing this out. We added a paragraph on novel insights in the discussion section, now in lines 585-597.

Reviewer #2:
The objectives are clear. The methodology does not entail testing a hypothesis and as such this is not applicable. The study design is appropriate and population well defined. Sample size calculations are also not necessary - point of theoretical saturation was used (though this is not referenced). No ethical concerns.
Thank you, we added information on theoretical saturation, now in lines 206-208.

**Results:**
-Does the analysis presented match the analysis plan?
-Are the results clearly and completely presented?
-Are the figures (Tables, Images) of sufficient quality for clarity?

Reviewer #1:

1. **Presentation and References**
   - Review and justify the necessity of each figure.

Thank you very much for pointing this out. We deleted Figure 1 as all the relevant information was already mentioned in the text now in lines 355-358.

- You don't seem to cite your previous qualitative research on vaccines.
Thank you, we added this information, now in lines 146-148.

2. **Analytical Depth**
- Narrow the research focus to provide more nuanced and in-depth analysis.
Thank you for pointing this out. We deleted the chapter "preventive measures of YF" and focused more on community perceptions and treatment practices.

- Strengthen the connection between community perceptions and actual treatment practices.
Thank you for pointing this out. We strengthened this connection and added an entire subchapter called "*Influence of disease perception on treatment-seeking behavior*", now in lines 496-541. We added the information on personal experience, now in lines 474-494. We added another subheading "*Healthcare accessibility and trust in medical care*" now in line 543.

Reviewer #2:
- The organization of the themes requires some attention - see the attached pdf
Thank you, we restructured the results accordingly. Please, see the specific comments for details.

**Conclusions:**
-Are the conclusions supported by the data presented?
-Are the limitations of analysis clearly described?
-Do the authors discuss how these data can be helpful to advance our understanding of the topic under study?
-Is public health relevance addressed?

Reviewer #1:
- The manuscript provides insights into community perceptions and treatment practices of yellow fever in Uganda. While the study offers valuable perspectives, the research would benefit from a more focused approach, concentrating on a specific core research question to enhance readability and impact.
Thank you, we used a more focused approach and clarified the conclusion, now in lines 746-752.

Reviewer #2:
- The conclusions are relatively sound though I suggest that they could extended somewhat to discuss how they can be applied.
Thank you for pointing this out. We made the conclusion more applicable, now in lines 746-752.

**Editorial and Data Presentation Modifications?**
Use this section for editorial suggestions as well as relatively minor modifications of existing data that would enhance clarity. If the only modifications needed are minor and/or editorial, you may wish to recommend "Minor Revision" or "Accept".

Reviewer #1: (No Response)
Reviewer #2: (No Response)

**Summary and General Comments:**
Use this section to provide overall comments, discuss strengths/weaknesses of the study, novelty, significance, general execution and scholarship. You may also include additional comments for the author, including concerns about dual publication, research ethics, or publication ethics. If requesting major revision, please articulate the new experiments that are needed.

Reviewer #1: (No Response)

Reviewer #2:

- A very worthwhile analysis of an under-studied area - although the implications of the gap between data collection and submission could be more clearly addressed.
Thank you, we added this important information more clearly in the limitations, now in lines 734-736.

- Some improvements could be made in the communication of the findings, through adjusting the structure of the results section - see the annotated pdf.
Thank you, we changed it accordingly. Please, see the point-by-point response at the specific comments.

**Specific comments- pdf**

Methods

- Lines 169-171: Please explain why this was the case: „In addition, participants had to be vaccinated during the aforementioned emergency immunization campaigns; therefore, they should have been exposed to YF awareness campaigns, which were part of the vaccination mobilization. Elderly men and elderly women were included at both study sites. "
Thank you, we explained it further in the lines 171-177.

- Lines 189: "We applied the COREQ guidelines to ensure data quality and credibility." COREQ us about reporting though.
Thank you for pointing this out. We changed it accordingly in the lines 194 and added more information on data quality and credibility in lines 195-208.

Results

- Line 229: Perceptions of Yellow fever: This section seems to bring together different elements, including understanding of severity and the awareness campaigns - I suggest that it be reorganized into the subsequent sections.
Thank you for pointing this out. We organized it into the following subheadings "*Perceptions of disease severity*" (now in lines 246-274)," *Perceptions of disease etiology*" (now in lines 276-309), "*Perceived cause of YF outbreaks*" (now in lines 311-326), and "*Influence of health education campaigns*" (now in lines 328-352).

- Line 232-234: "The information reaching remote communities through awareness campaigns portrayed it as a deadly disease yet failed to provide sufficient information." According to whom?
Thank you, we clarified it, now in line 249.

- Line 396: "Personal experience with YF ". I would suggest either weaving this through the other sections or presenting specific cases - in this way you don't really do one or the other.
Thank you for pointing this out. We deleted the heading and included relevant information into other chapters, such as in "treatment-seeking behaviors" (now in lines 474-494), in the chapter "Perceptions of disease severity" (now in lines 271-

274), in “Perceptions of disease etiology” (now in lines 290-303), and in the chapter “Challenges in obtaining a diagnosis” (now in lines 457-460).

- Line 445: “Info box 1: Community perception and diagnostic pathway of YF cases in an outbreak-445 affected community in 2016.” Why is this in a separate box? Would it fit with the other sections...i.e. it potentially as a normal section before you start talking about therapies?
  Thank you, we deleted the Info box 1 and created a normal section called “Challenges in obtaining a diagnosis”, right before the treatment-seeking behavior chapter, now in lines 436-460. Furthermore, we transferred information into better suiting sections, such as into “Perceptions of disease etiology “, now in lines 305-309, and “Influence of health education campaigns” now in lines 337-340.

Discussion

- Line 573: „This observation reflects a basic understanding of health risks“. I am not sure that this is the most appropriate term.
  Thank you for pointing this out. We changed the term „basic“ to general, now in line 613.

- Line 591-592: “Overall, to develop more effective public health interventions, it is crucial to understand these perceptions of YF transmission.“ This seems somewhat teleological...and I am not sure that you need to justify the study at this stage.
  Thank you, we deleted this sentence.

- Lines 648-658: This section is not well linked with the findings.
  Thank you for pointing this out, we deleted this paragraph.

- Lines 669-675: I think that you can say a bit more here in terms of how the communities that are the targets of such information campaigns could be involved in their design in implementation.
  Thank you. We added more information, now in lines 702-709.

Strengths and limitations

- Lines 684-685: „A strength of this manuscript is our exploration of the perceptions of vulnerable groups in rural and remote villages regarding disease perception and treatment practices for YF.“ I am not sure that this is a strength ...or at least you should frame it more in terms of the following sentence...i.e. its uniqueness.
  Thank you, we changed it accordingly. Now in line 718.

- Strengths & limitations: An important limitation seems to be the time that has passed since the data were collected.
  Thank you, we added this information in lines 734-736.

Conclusion

- Lines 710-711: “ Understanding the cause of the outbreak and its transmission modes is crucial for taking appropriate public health actions.“ I am not sure that this quite follows form the previous sentence...at least when framed in as it is in terms of public health actions.
  Thank you for pointing this out. We improved our conclusion, now in lines 746-752.

We updated the terminology throughout the manuscript, replacing the outdated term “elderly men and women” with “older adults” and, where relevant, “older men” and “older women” to ensure respectful and current language.
